# The experience of living with Niemann–Pick type C: a patient and caregiver perspective

**DOI:** 10.1186/s13023-023-02741-2

**Published:** 2023-05-20

**Authors:** Emma Golden, Raquel van Gool, Mariesa Cay, Benjamin Goodlett, Amanda Cao, Walla Al-Hertani, Jaymin Upadhyay

**Affiliations:** 1 Department of Anesthesiology, Critical Care and Pain Medicine, Boston Children’s Hospital, Harvard Medical School, Boston, MA 02115 USA; 2 Department of Neurology, School of Mental Health and Neuroscience, Maastricht University Medical Center+, Maastricht, The Netherlands; 3 Division of Genetics and Genomics, Boston Children’s Hospital, Harvard Medical School, Boston, MA USA; 4 Department of Psychiatry, McLean Hospital, Harvard Medical School, Belmont, MA USA

**Keywords:** Niemann–Pick disease type C, Central nervous system, Cognition, Motor, Caregiver, Focus group

## Abstract

**Background:**

Niemann–Pick disease type C (NPC) is a rare inherited lysosomal storage disease typified by accumulation of cholesterol and other lipids in late endosomes/lysosomes, thereby resulting in a spectrum of neurological, psychiatric, and systemic symptoms (notably liver disease). Though it is well-known that NPC exacts a physical and emotional toll on both patients and caregivers, the burden of NPC can vary between patients, while the challenges of living with NPC can evolve over time (i.e., from time of diagnosis to the present day). To further grasp patient and caregiver perceptions and experiences with NPC, we carried out focus group discussions with pediatric and adult individuals with NPC (N = 19), with partial or full representation of the patient by their caregiver. Furthermore, we utilized our NPC focus group discussion to provide guidance on study design parameters and feasibility of prospective investigations aiming to characterize the central manifestations of NPC using neuroimaging, specifically, magnetic resonance imaging (MRI) methodology.

**Results:**

Focus group discussions revealed that neurological signs, including declining cognition, memory loss, and psychiatric symptoms, as well as increasingly impaired mobility and motor function, are among the most pressing past and current concerns for patients and caregivers. Moreover, several participants also expressed concern over a loss of independence, social exclusion, and uncertainty for what the future holds. Caregivers described the challenges that participation in research poses, which included logistical difficulties mainly due to traveling with medical equipment and the need for sedation in a minority of patients when undergoing MRI.

**Conclusions:**

The findings derived from focus group discussions highlight the outstanding challenges that NPC patients and their caregivers face daily, while also providing direction on the potential scope and feasibility of future studies focusing on the central phenotypes of NPC.

## Background

Niemann–Pick disease type C (NPC) is a rare progressive genetic disease caused by mutations in *NPC1* or *NPC2* [1, 2], which encode essential lysosomal proteins involved in intracellular transport and lipid metabolism [3]. Disruption of these intracellular processes lead to cholesterol and lipid accumulation in late endosomes/lysosomes, resulting in a spectrum of clinical manifestations that ranges from a fatal disorder within the first few months of life to an adult-onset neurodegenerative disorder [1–3]. NPC is generally classified into four categories based on age at onset: early-infantile, late-infantile, juvenile, and adolescent/adult-onset [4]. Age at onset of neurological manifestation is a predictor of disease progression, which widely varies between individuals [3, 5]. Infantile onset of NPC leads to a more rapidly progressing fatal disorder with primarily neuromotor symptoms, while the adult-onset form slowly progresses with notable decline in cognitive and psychiatric symptoms [3, 6–9].

Treatment of NPC commonly consists of a multi-disciplinary therapeutic approach focused on symptom management and administration of substrate reduction therapy [10]. Currently, Miglustat is approved for use in NPC in several countries, but not by the Food and Drug Administration (FDA) [10–12]. Other therapeutics currently under investigation include IV HPβCD (phase III; NCT04860960) and Arimoclomol (expanded access program; NCT004316637). As there are no FDA approvals for NPC at this time, nor an available cure, it is imperative to understand the full impact and burden of NPC to gain insight into patient and caregiver needs; information which can be used to optimize disease management and better define the scope of future clinical research studies.

Patients with NPC often experience a broad range of neurological and psychiatric signs and symptoms [8, 9, 13, 14]. Moreover, the weight that these symptoms carry often vary between NPC patients and may also evolve over time, bringing on new challenges and concerns for both the patient and caregivers. To further grasp the impact of living with NPC, we recently conducted focus group discussions and qualitative interviews involving a diverse sample of pediatric and adult NPC patients and their caregivers. NPC patients who participated in the interviews possessed a range of disease severity, central manifestations, and variable baseline characteristics such as age of symptom onset, age of diagnosis, current treatment, and geographical location. The objective of the current study was two-fold. Objective 1 was to gauge the occurrence and impact of different sets of neurological and psychiatric symptoms on patients’ lives both in the past (i.e., at the time of symptom onset or NPC diagnosis) and in the present. However, when asked to voice their respective concerns and experiences with NPC, patients and caregivers were not instructed to restrict the discussion to neurological and psychiatric symptoms. Objective 2 was to garner information to shape the design and scope of future clinical research studies, where central manifestations of NPC would be probed using a multidisciplinary approach that incorporates structural and functional magnetic resonance imaging (MRI). Here we aimed to identify any potential barriers that would limit study participation or would enhance the experience for the patient and family during their involvement in the study.

## Results

### NPC patient demographics

A total of 19 patients were recruited and enrolled for the interviews, of whom 17 were either fully or partially represented by their caregiver. One patient, represented by their caregiver, was deceased at the time of data collection. The study population consisted of 6 patients who received their diagnosis before age 15 and 13 patients who received a diagnosis later in life. Table 1 provides an overview of the sample population, which consisted of multiple sibling pairs.

**Table 1:** Overview of NPC patients

Patient	Age	Gender	Age at symptom onset	Age of diagnosis	Focus group responder
1	29	M	–	14	P + C
2	19	M	3	10	P + C
3	24	M	–	22	P + C
4*	30	F	Mid-to-late 20’s	27	P + C
5	63	F	47	59	P + C
6*	33	M	–	30	P + C
7	43	F	–	39	P
8	47	M	–	46	P
9*^#^	35	M	20–21	30	C
10*^#^	32	M	23–24	28	C
11^a^*	33	F	7–8	29	C
12	15	M	7–8	14	C
13	25	M	20–21	24	C
14^##^	39	F	18	36	C
15*^##^	34	M	–	30	C
16*^##^	28	M	22–23	25	C
17^###^	24	M	–	10	C
18^###^	21	F	–	7	C
19	16	M	–	14	C

C, caregiver; P, patient

*Patients were misdiagnosed with schizophrenia spectrum disorder, bipolar disorder, and other psychiatric illnesses for multiple years in some cases, which resulted in substantial delays in diagnosis and appropriate treatment

^#^Siblings set 1

^##^Siblings set 2

^###^Siblings set 3

^a^Patient deceased

### Objective 1: neurological and psychiatric signs and symptoms of NPC

**Fig. 1:**
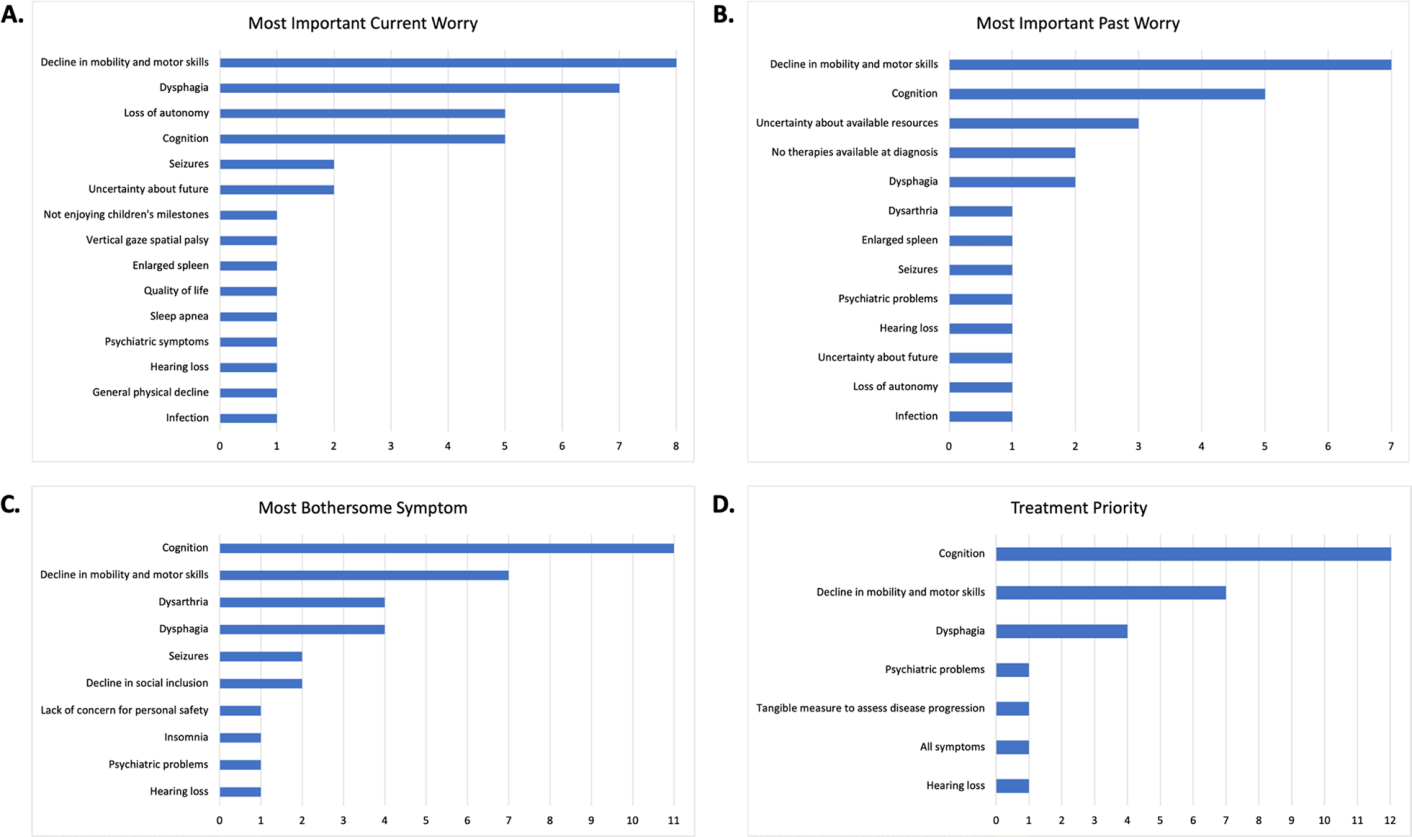
Ranking of symptom worry in the present and past. **A** Most important current worry is a decline in mobility and motor skills, which encompasses concerns about gait and balance, risk of falling, and ataxia. Dysphagia is the second-most important current worry, followed by loss of autonomy and cognition. **B** Most important past worry was a decline in mobility and motor skills. Cognition is mentioned as second-most important past concern. Although N = 7 patients experienced psychiatric symptoms in the past, concerns about psychiatric problems were not most important. **C** Most bothersome symptom in the present is cognitive difficulty. Decline in mobility and motor skills is the second-most bothersome symptom. **D** Cognition is most often mentioned as a treatment priority

Qualitative analysis of patient or caregiver responses demonstrated a spectrum of neurological and psychiatric symptoms both at the time of diagnosis and during study participation (Table 2). Frequently reported neurological symptoms included memory loss, executive dysfunction, and psychosis-related symptoms (e.g., delusions, hallucinations, and paranoia), while concerns about the decline in sensorimotor functioning included ataxia, dysphagia, dysarthria, concerns about falling, difficulty balancing, and gait impairment, among others. Cognitive difficulties (loss of working memory or ability to plan simple tasks) or psychosocial challenges (difficulties with verbal communication or feelings of isolation), as well as life-threatening symptoms (dysphagia and motor-related difficulties), were both important current and past concerns of patients and caregivers (Fig. 1). Caregivers communicated worry about symptoms related to patients’ physical well-being, with increased risk of dangerous situations and risk of falling most often reported. Patients who could communicate clearly with the staff described worry about symptoms that impact their participation in daily life, such as hearing loss, ataxia, and cognitive impairment. For NPC patients whose symptoms were noticed before the age of 15 (N = 6), seizures, sleep apnea, and dysarthria were reported to be a significant burden both in the past and at the time of the interview, with one patient experiencing multiple seizures per day. As reflected in Table 2, in the current cohort, the occurrence of seizures was more frequently reported for NPC patients diagnosed in childhood vs. adulthood, while the presence of psychiatric manifestations appeared more common in patients who were diagnosed later in life as adults. In Tables 2, 3 and 4, patients are listed according to age of diagnosis.

**Table 2:** Overview of interview responses

Patient ID (age at diagnosis)	Most important current worry	Most important past worry	Most bothersome symptoms	Treatment priority
18*** (7)	Seizures	No therapies available at diagnosis	Cognition	Cognition
2 (10)	Dysphagia, infection, decline in mobility	Dysphagia, infection, decline in mobility	Dysarthria, dysphagia, comprehension difficulty	Dysphagia (cognition is stable)
17*** (10)	Dysphagia and mobility	No therapies available at diagnosis	Seizures, dysphagia, mobility	Motor functions and aspiration
12 (14)	Sleep apnea, seizures, cognition	Seizures	Seizures and loss of physical function	Cognition and physical functioning
19 (14)	Enlarged spleen, vertical gaze spatial palsy; parent: not enjoying child’s milestones	Cognition	Dysarthria, cognition	Cognition
1 (14)	Memory loss	Motor deficits	Cognition and motor deficits	Memory and cognition
3 (22)	Unsure	Unsure	Tripping and falling	Unsure
13 (24)	Cognition	Cognition	Cognition	No tangible way available to measure improvement/decline
16** (25)	Autonomy	Uncertainty about available resources	Cognition	Cognition and motor functions
4 (27)	Motor deficits, dysphagia, autonomy, uncertainty about future	Motor deficits, dysphagia, autonomy	Cognition and social decline	Cognition
10* (28)	Psychiatric and behavioral problems (paranoia, delusional thinking, anger, frustration), dysphagia	Psychiatric problems	Psychiatric problems, cognition	Psychiatric problems, cognition, dysphagia
11^a^ (29)	N/A	Cognition	Social	All symptoms
6 (30)	Autonomy, falling, uncertainty about future	Uncertainty about future	Insomnia, cognition	Cognition
15** (30)	Autonomy	Uncertainty about available resources	Motor deficits	Cognition and motor functions
9* (30)	Cognition, fine motor skills, dysphagia	Memory and balance	Cognition	Cognition
14** (36)	Dysphagia, motor deficits, quality of life	Uncertainty about available resources	Dysphagia	Cognition and motor functions
7 (39)	Autonomy and general physical decline	Ataxia	Gait, ataxia, dysarthria	Ataxia
8 (46)	Hearing loss and cognition	Hearing loss and cognition, enlarged spleen	Hearing loss and cognition	Hearing loss and cognition
5 (59)	Patient: gait and balance; caregiver: motor deficits and dysphagia	Patient: gait and balance; caregiver: dysarthria and gait	Caregiver: dysarthria, lack of concern for safety	Patient: gait and balance; caregiver: dysphagia

*Sibling set 1, Patients 9 and 10

**Sibling set 2, Patients 14, 15, and 16

***Sibling set 3, Patients 17 and 18

^a^Patient deceased

**Table 3:** Logistical challenges

Patient (age at diagnosis)	Logistical difficulties related to study participation
18 (7)	Caregivers unable to travel
2 (10)	No
17 (10)	Travel difficulties with medical equipment
12 (14)	No
19 (14)	No
1 (14)	No
3 (22)	No
13 (24)	No
16 (25)	No
4 (27)	No
10 (28)	No
11^a^ (29)	Travel difficulties
6 (30)	No
15 (30)	Caregiver requested transportation and lodging to be organized
9 (30)	No
14 (36)	Caregiver requested transportation and lodging to be organized
7 (39)	No
8 (46)	Caregiver requested more financial compensation
5 (59)	No

Sorted based on age of diagnosis, in ascending order

^a^Patient deceased

**Table 4:** Challenges associated with undergoing MRI procedures

Patient (age at diagnosis)	Potential difficulties undergoing MRI
18 (7)	Requires someone in the scanning room
2 (10)	Requested someone in the scanning room
17 (10)	Cannot lie still, would need sedation
12 (10)	Anxiety, possibly needing sedation
19 (14)	No
1 (14)	No
3 (22)	Potentially shortened scan, involuntary movements
13 (24)	No
16 (25)	Might need music or a movie
4 (27)	No
10 (28)	No
11^a^ (29)	Did not pose difficulties in the past
6 (30)	No
15 (30)	Claustrophobia
9 (30)	No
14 (36)	Involuntary movements
7 (39)	No
8 (46)	No
5 (59)	No

Sorted based on age of diagnosis, in ascending order

^a^Patient deceased

Caregivers, specifically parents, communicated that their children experienced learning challenges (e.g., difficulties with reading), mobility deficits (e.g., clumsiness), and behavioral problems (e.g., anger or quick temper) during childhood or adolescence, and only later received an NPC diagnosis. Caregivers found it difficult to pinpoint when certain symptoms were initially present, as in some cases the signs and symptoms were initially subtle, non-specific, and often remained undetected for long periods of time (e.g., multiple years). Substantial progression of symptom severity and clinical presentation prompted caregivers and patients to seek further clinical care and insights; however, misdiagnosis (i.e., schizophrenia spectrum disorder) or late diagnosis of NPC were common. One caregiver expressed that even when an adult sibling was diagnosed with NPC, there was and remains denial of having NPC, and therefore a resistance to undergoing specific treatment for NPC or incorporating simple changes in lifestyle. Moreover, adult-onset participants were reported to experience psychiatric and behavioral problems, impacting their ability to complete their education, or introducing career-related challenges. Some patients were reported to have withdrawn from their studies, including college, and in one case military service due to experiencing psychosis. One NPC patient has unfortunately struggled to keep employment as a result of behavioral problems or difficult interactions with other employees. For adult NPC patients who can hold some type of steady employment, caregivers have noted that doing so has provided a sense of routine, sense of purpose, and valuable social interactions.

### Objective 2: patient and caregiver guidance on prospective study design focus and feasibility

Patients expressed interest and enthusiasm regarding participating in a study focusing on central manifestations of NPC. Importantly, the majority of participants noted a willingness to travel to our study site in Boston, Massachusetts (Table 3). Although some participants informed study staff on logistical challenges they would likely face and that they would require resources to overcome these hurdles. Interestingly**,** patients who were non-ambulatory and their caregivers indicated that difficulties with mobility would not hinder their study participation. Some caregivers indicated that travelling comes with many challenges, particularly for those with more severe physical symptoms, and recommended to organize transportation and investigate specific accommodations for study participants well ahead of time. Individuals with a later age of diagnosis more frequently requested assistance with travel and logistics. This may be attributed to more advanced disease progression.

To investigate the feasibility of NPC patients to undergo non-contrast structural and functional MRI of the CNS without use of sedation, the interview also included questions and discussions about the possible difficulties that may present when undergoing potential study-related procedures. Table 4 provides an overview of the challenges that may come with MRI studies for the current NPC cohort. Although many of the patients reported experiencing no problems undergoing prior MRI scanning, some patients were reported to present with claustrophobia, anxiety, or involuntary movements. Individuals with childhood diagnoses most frequently reported potential difficulties with scanning, but this is likely attributed to the challenges inherent to obtaining quality MRI scans in a pediatric population [15]. However, most caregivers and patients noted that temporary feelings of anxiety might be overcome with having someone close by in the MRI scanner room or when possible, having music played while the patient is in the scanner.

## Discussion

To further our understanding of the impact of NPC, particularly the central phenotypes of the disease, qualitative interviews were held with a diverse spectrum of NPC patients and their caregivers. Our qualitative discussions with NPC patients and families centered around two objectives. The first objective was to assess the occurrence and variable manifestations of the signs and symptoms of NPC, as well as the impact they have on the lives of patients and caregivers. Relatedly, the second objective was to gain a deeper understanding of the various facets of NPC that are critical from the perspective of NPC patients and caregivers, as well as the challenges NPC families face when participating in clinical research. The information gathered from pursing these two objectives can be used in order to better shape the design of future studies on the central manifestations of NPC, specifically those incorporating structural and functional MRI.

The impact NPC has on the lives of affected individuals is variable but nonetheless deeply profound. Cognitive difficulties (memory deficits or executive dysfunction), motor function impairment, psychosocial challenges (verbal communication difficulties, loss of autonomy, and social exclusion), and other specific symptoms such as dysphagia, ataxia, dysarthria, and hearing loss were among the most important current and past concerns for patients and caregivers. These were also reported to be the most bothersome symptoms, as well as the most prioritized symptoms in need of treatment from the perspective of patients with NPC or their caregiver. Caregivers, primarily parents of individuals with NPC, noted that the outcome of NPC-related signs and symptoms combined with the fact that there remain limited treatment options for patients has brought on emotional difficulties that are perhaps unappreciated from an outside perspective. Mainly, it is not solely that there is a loss of cognitive or motor ability, but perhaps equally important, these and other central manifestations of the disease hinder a parents’ experience of seeing their children reach certain physical, educational, or social milestones in life or attain a basic level of independence or autonomy. In cases where a family consists of multiple NPC patients, the burden of the disease is understandably compounded and high for the caregiver as the clinical presentation and daily or long-term needs for each patient can vary.

In addition to concerns that arose and continue to persist following their NPC diagnosis, many individuals acknowledged their experiences and frustrations with delays in a correct NPC diagnosis. For the late-infantile and juvenile forms of NPC, diagnosis is, on average, delayed by ~ 4.1 years [14]. In our representative population, participants experienced, on average, a 7.6-year delay between symptom onset and receiving an NPC diagnosis. These lengthy diagnostic delays not only lead to years of uncertainty and misdiagnosis for families and individuals, but potentially result in lost opportunities for earlier disease management or targeted, symptom-specific treatment plans [4]. Participants cited heterogeneous presentation, early non-specific symptoms, and lack of clinician awareness about NPC as diagnostic hindrances. Patients in the study presented with a wide variability in symptom manifestation. Even among sibling groups (sibling set 1: patients 9 and 10; sibling set 2: patients 14–16; sibling set 3: patients 17 and 18), there exist diverse clinical presentations (Table 2). The lack of correlation between relatedness and symptom onset or disease severity is especially notable in the sibling group of patients 14–16. Patient 14 is non-ambulatory, non-verbal, and requires a GI-tube for feeding. Their siblings, in contrast, are ambulatory, verbally communicative, and eat without assistance, and instead experience psychiatric manifestations and memory impairment. Several participants noted a childhood diagnosis of attention-deficit hyperactivity disorder, difficulties in school, and poor memory at a young age as early clinical or behavioral indicators that went largely unrecognized or, at the very least, under-appreciated by caregivers, clinicians, or teachers. Others experienced gait impairment, uncontrollable twitches, and issues with tripping, falling, and poor balance. Additionally, multiple participants had severe psychiatric manifestations and were misdiagnosed with schizophrenia spectrum disorder, bipolar disorder, or other psychiatric syndromes, causing months-to-years long delays in proper diagnosis and likely exposure to unnecessary or non-efficacious treatments. A robust and unfortunate example of such a case is patient 11, who did not receive a correct NPC diagnosis for more than a decade after the parent noted symptom onset. These experiences of misdiagnoses or delays in diagnoses as reported by the patients and caregivers underscore the ongoing need for timely diagnosis and early intervention.

Several tools are available to aid clinicians in identifying the signs and symptoms of NPC and to measure overall disease burden and treatment efficacy. Clinical measures include the NPC Suspicion Index Tool [16], NPC Clinical Severity Scale (NPCCSS) [17], and the Assessment and Rating of Ataxia (SARA) scale [18, 19]. In addition to these benchmark clinical instruments, circulating biomarkers such as bile acids (of 3β-sulfooxy-7β-N-acetylglucosaminyl-5-cholen-24-oic acid and its glycine- and taurine-amides), and lysoSM-509 (N-palmitoyl-O-phosphocholineserine [PPCS]) are used for NPC diagnosis [20–22]. However, their ability to detect central therapeutic responses remains ambiguous [22, 23]. While benchmark clinical tools assess for cognition and decline in mobility [10, 24], multiple participants expressed a desire for a more tangible measure of disease progression and/or therapeutic response. Currently in NPC, there are no validated biomarkers that closely associate with central nervous system (CNS) manifestations or disease progression.

Despite a strong desire and interest towards participating in research studies, NPC patients and caregivers may hesitate to become involved in clinical investigations, particularly those encompassing potentially demanding methods such as MRI or other forms of neuroimaging. On one hand, identifying and validating new tools or approaches that can accurately inform on CNS disease in NPC are critically needed, while on the other hand, newly developed tools should be feasible to employ across a broad spectrum of patients with NPC who can vary according to age, disease severity, and overall clinical presentation. From our focus group discussions, there was an openness to participate in a wide range of studies. However, barriers to do so were mainly logistical in nature for a subpopulation of the current NPC cohort. Caregivers or NPC patients themselves reported that transportation comes with many challenges, due largely in part to patients travelling with medical equipment, being non-ambulatory, or requiring some level of supervision from a caregiver due to cognitive or motor-related impairments. However, provided that logistical support and resources (for patient and caregiver) are well-organized ahead of time by members of the study team, study participation to even distant study sites were not considered a major hurdle. With regards to undergoing MRI procedures, most focus group participants noted no prior issue undergoing imaging, while some noted that sedation was necessary during MRI and at earlier stages of the disease when the NPC patient was in a less stable mental and physical state. Experiencing claustrophobia, anxiety, or involuntary movements in an MRI environment was noted by several caregivers, but most expressed that this challenge could be overcome using non-sedative means (i.e., music, having someone within the MRI room, or undergoing some preparations in a mock MRI environment).

The presence of cognitive impairment was a very common symptom that diminished the quality of life for NPC patients or represented a common source of concern for all study participants. This was true independent of past or current status of disease or symptom severity. Although many individuals in our cohort were considered to have adult-onset NPC, for which cognitive impairment is often present [6, 8], concerns related to cognition also extend to younger patients and their caregivers. Both patients and caregivers expressed worry about the decline of cognitive abilities and described this loss of function as greatly contributing to reductions in independence. Understanding the biobehavioral basis of cognitive dysfunction in NPC is paramount, but also a challenging task in NPC. For example, although there are numerous neuropsychological tests such as the N-Back, Stroop, or Go-No Go tasks, which are frequently employed as strictly behavioral methods or in combinations with fMRI data acquisition, their incorporation in an NPC study may be challenging as the cognitive capabilities and a basic understanding of task procedures can be difficult for many low functioning NPC patients with progressive disease [25–27]. Therefore, an implementation of behavioral assessments and batteries such as the Kaufman Assessment Battery for Children (KABC-II) or the Vineland Adaptive Behavior Scale should be considered as these tools are better suited for individuals with intellectual or developmental disabilities [28]. Additionally, use of low burden, resting-state fMRI procedures might also be considered for studies aiming to uncover and characterize functionally affected CNS circuitry in NPC patients [29–33].

Given the overall framework of focus groups, discussions occurring in this context generally offer an opportunity for study teams to perform a deep dive into severe, complex, and heterogeneous diseases such as NPC. Dialogue occurring during focus groups can facilitate an engaging and flexible conversation, where patients and caregivers can communicate various aspects of living with a chronic disease without the constraints of, for example, clinical questionnaires or behavioral tasks. Nonetheless, this focus group consisted of multiple limitations. Firstly, information gathered from this focus group is primarily qualitative or semi-quantitative in nature at best. In our experience, participants were open and forthcoming, but we recognize that some aspects of living with NPC or having a child or partner with NPC may be emotionally challenging, making certain elements of NPC difficult to articulate. Secondly, the participants in our focus group had variable access to informed clinical care, either prior to or during the time of diagnosis, as well as presently; some individuals had access to physicians and clinical care staff intimately familiar with NPC, while others in a different geographical setting had more limited access. This variable access to healthcare relevant to NPC may have contributed to the heterogeneity of concerns raised by patients and caregivers. Unfortunately, the current study could not disentangle the exact causes underlying the issues raised during the focus group. Finally, the sample size of this study (N = 19) was also moderate, which limited our ability to make distinctions between, for example, early vs. late diagnosis. Yet, this study provides the impetus for designing studies to identify certain phenotypes and further developing more detailed hypotheses to test in future investigations.

While the current study findings were derived from NPC patients and families, there are indeed parallels with other rare disease populations in terms of the impact that the illness has on primary caregivers [34–37]. Given the wide range of emotional, physical, and logistical demands that family members or caregivers often face daily, there are universal problems imposed on the caregiver that reduce their overall health-related quality of life, ability to pursue personal goals and objectives, or productivity. Moreover, when planning prospective investigations involving NPC patients and other rare disease populations, the burden on caregivers should also be considered and steps taken to minimize challenges these individuals may face throughout the study.

## Conclusions

In conclusion, this study revealed that, while NPC has highly heterogeneous manifestations, there are also many parallels regarding which aspects of the disease have impacted the families across the diagnostic timeline; many concerns remain consistent between initial diagnosis and present day. Patients and caregivers most frequently worry about loss of cognitive abilities, loss of motor skills, and the challenges these impairments pose, both in day-to-day life and in a more intangible sense, such as missing an opportunity to experience certain developmental or social milestones and attain independence. From our focus group, we also determined that studies should be multidisciplinary in nature to best understand the variable domains of NPC. While some methods may present challenges, such as non-contrast, non-sedated MRI, they are likely feasible and informative to disentangle aberrant neurocircuitry characteristics in NPC.

## Methods

This focus group and qualitative interview study was approved by the Boston Children’s Hospital (BCH) Institutional Review Board. Patient outreach was aided by the National Niemann–Pick Disease Foundation (NNPDF). Additional participants consisted of NPC patients treated at Boston Children’s Hospital. Potential NPC patients and families, identified via referrals through the NNPDF or from physicians treating individuals with NPC at Boston Children’s Hospital, were sent a brochure summarizing the purpose and scope of the study, and were extended the option of participating in a focus group or one-on-one interviews. All study participants, which included NPC patients, family members, or legal guardians gave verbal informed consent prior to the initiation of any discussions. For patients with NPC with severe disability, verbal consent was obtained from the caregiver(s) and on the patients’ behalf. All participants were made aware that they were not required to answer any questions that made them uncomfortable and could take a break during the focus group at any time. All individuals who took part in either focus group discussion (i.e., group 1: patients 9–13 and group 2: patients 14–18) were given the option to reach out to study staff offline to communicate any information, further questions, or concerns in a non-group setting; however, no participant took this latter option.

### Study design

Interviews were held both virtually over a secure BCH-approved Zoom video conference call as well as in person. Two separate, virtual focus groups were conducted in which the caregivers represented the patients (group 1: patients 9–13 and group 2: patients 14–18). All other interviews were conducted one-on-one with the patient/caregiver, in-person (patients: 1–8) and virtually (patient: 19). Individual interviews were conducted to accommodate the patients’ and families’ schedules. Both focus groups and all one-on-one interviews were semi-structured. While the discussion incorporated a set of pre-determined questions, participants were welcomed to expand where they felt necessary and speak outside the confines of the questions. All interviews revolved around the following pre-established questions:

1. What is your (referring to the individual with NPC) age and gender?
2. Were you diagnosed before or after the age of 18?
3. What parts of your disease worry you most now?
4. What parts of your disease have worried you the most in the past?
5. Are there specific symptoms that bother you more than others?
6. Which elements of your disease are important to get treated sooner rather than later?
7. Would you be willing to travel to Boston, MA for a study?
8. Would you be willing to undergo a brain MRI that lasts for 50–60 min (with no injection)?
9. If 50–60 min is too long, what is the longest you would spend in an MRI?
10. Would you be okay with having your blood drawn?
11. Is there anything a study team or study team member can do to make being a part of this study easier for you?

Responses to each question were, when possible, given by the participants with NPC. However, in many cases, due to communication difficulties or cognitive symptoms, the caregiver, primarily a parent, sibling, or partner, represented the patient. There were no limitations set in terms of who could or could not participate. On average, interviews lasted for approximately 60 min with slightly longer time periods needed for the larger participant groups. The largest focus group consisted of 5 NPC families. Participants were told that a prospective study may take place at Boston Children’s Hospital and may involve a blood draw and a 50–60-min non-contrast MRI scan. Focus groups and individual interviews were held with the intention of assessing the feasibility of an MRI study in an NPC patient population. Our hope is that the information gained will allow for broader accessibility and participation in future NPC MRI studies.

## Data Availability

The datasets generated and/or analyzed during the current study are not publicly available due institutional regulations but are available from the corresponding author on reasonable request.

## Abbreviations

BCH: Boston Children’s Hospital
C: Caregiver
CNS: Central nervous system
fMRI: Functional magnetic resonance imaging
NPC: Niemann–Pick type C
NNPDF: National Niemann–Pick Disease Foundation
MRI: Magnetic resonance imaging
P: Patient
